# In-room treadmill exercise stress cardiac magnetic resonance in patients with suspected ischemic heart disease

**DOI:** 10.1186/1532-429X-11-S1-O40

**Published:** 2009-01-28

**Authors:** Subha V Raman, Mihaela Jekic, Jennifer A Dickerson, Eric L Foster, Orlando P Simonetti

**Affiliations:** Ohio State University, Columbus, OH USA

**Keywords:** Cardiac Magnetic Resonance, Perfusion Imaging, Treadmill Exercise, Stress Cardiac Magnetic Resonance, Cine Cardiac Magnetic Resonance

## Objective

To implement and demonstrate the feasibility of in-room treadmill exercise stress perfusion and cine CMR in patients with suspected coronary artery disease (CAD).

## Background

Exercise is preferred to pharmacologic stress because it links physical activity to symptoms and ischemia and offers important information such as exercise capacity, blood pressure response, ECG changes, and the presence or absence of exercise-induced symptoms. The Bruce Treadmill Test is the most commonly-used protocol for cardiac stress testing in the US, with proven diagnostic and prognostic value. The lack of MRI-compatible exercise equipment has made pharmacological stress the only practical option for CMR stress testing to date. We implemented treadmill exercise stress cardiac magnetic resonance imaging (CMR) of both wall motion and perfusion in patients with suspected ischemic heart disease.

## Methods

A treadmill was modified by replacing all ferromagnetic components except the motor with non-magnetic equivalents. This enabled safe placement of the treadmill in the corner of the MRI room, approximately 2 m. from the patient table. Sixteen patients age 56 ± 8 years referred for stress SPECT were prospectively enrolled. Tc99m SPECT imaging was performed at rest; patients were then moved to the MRI suite for stress testing. Patients were positioned on the MRI table using a vacuum mattress to enable precise repositioning following treadmill exercise. Localizer scans followed by resting real-time cine CMR were performed, and then cine and perfusion scans were queued for rapid execution immediately following treadmill exercise. After removing the patient from the magnet, resting ECG was recorded and treadmill exercise commenced using the Bruce protocol. 12-lead ECG monitoring was performed throughout the treadmill test. At peak stress, Tc99m was injected and patients rapidly returned to their prior position in the magnet for post-exercise real-time cine followed immediately by multislice first-pass perfusion imaging with 0.1 mmol/kg IV gadolinium-based contrast using GRE-EPI TR/TE 5.8/1.2 ms, ETL 4 m matrix 160 × 96 and TSENSE acceleration factor of 2. The patient table was pulled out of the MRI system and patients remained supine on the patient table for 12-lead ECG monitoring during 5–10 min of recovery. The table was then returned to magnet bore for recovery cine and resting perfusion followed by delayed post-gadolinium imaging. Post-CMR, patients went to the adjacent SPECT lab for stress nuclear imaging. Five patients underwent coronary angiography. Images were reviewed blinded to other results.

## Results

**Figure 1:**
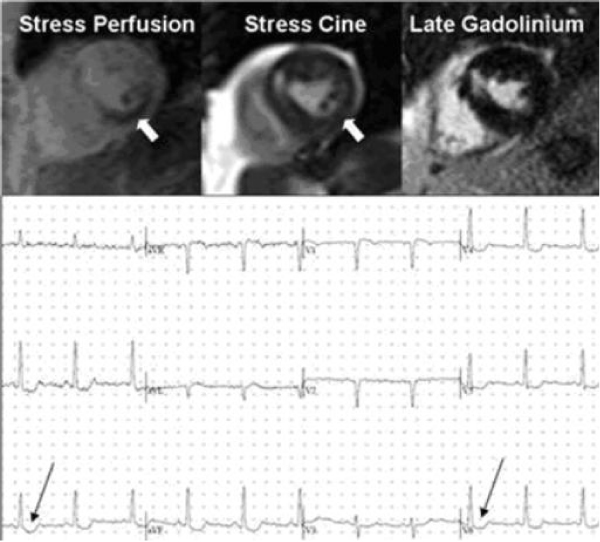
Figure 1

All patients completed the examination (Figure 1: ischemia example). Mean time to completion of cine MRI post-exercise was 73 ± 9 sec, and to completion of perfusion imaging 91 ± 6 sec. Accuracy in the five patients who underwent coronary angiography was 5/5 for CMR and 3/5 for SPECT. Follow-up at median of 60 days indicated freedom from cardiovascular events in 13/13 CMR-negative and 12/13 SPECT-negative patients.

## Conclusion

Exercise stress CMR including wall motion and perfusion inside the MRI room is feasible in patients with suspected ischemic heart disease. Preliminary results indicate favorable accuracy and prognostic value of this new stress imaging system compared to nuclear perfusion imaging. Further technical modifications are required to facilitate completion of cine and perfusion imaging within 1 minute of peak stress.

